# Gene editing monkeys: Retrospect and outlook

**DOI:** 10.3389/fcell.2022.913996

**Published:** 2022-09-08

**Authors:** Weizheng Liang, Junli He, Chenyu Mao, Chengwei Yu, Qingxue Meng, Jun Xue, Xueliang Wu, Shanliang Li, Yukai Wang, Hongyang Yi

**Affiliations:** ^1^ Central Laboratory, The First Affiliated Hospital of Hebei North University, Zhangjiakou, China; ^2^ Department of Pediatrics, Shenzhen University General Hospital, Shenzhen, China; ^3^ University of Pennsylvania, Philadelphia, PA, United States; ^4^ School of Future Technology, University of Chinese Academy of Sciences, Beijing, China; ^5^ Department of General Surgery, The First Affiliated Hospital of Hebei North University, Zhangjiakou, China; ^6^ Department of Pharmacology, Guangxi University of Chinese Medicine, Nanning, Guangxi, China; ^7^ State Key Laboratory of Stem Cell and Reproductive Biology, Institute of Zoology, Chinese Academy of Sciences, Beijing, China; ^8^ Institute for Stem Cell and Regeneration, Chinese Academy of Sciences, Beijing, China; ^9^ Beijing Institute for Stem Cell and Regenerative Medicine, Beijing, China; ^10^ National Stem Cell Resource Center, Chinese Academy of Sciences, Beijing, China; ^11^ National Clinical Research Centre for Infectious Diseases, The Third People’s Hospital of Shenzhen and The Second Affiliated Hospital of Southern University of Science and Technology, Shenzhen, China

**Keywords:** nonhuman primates, gene editing, rhesus monkeys, cynomolgus monkeys, CRISPR/Cas9, human diseases

## Abstract

Animal models play a key role in life science research, especially in the study of human disease pathogenesis and drug screening. Because of the closer proximity to humans in terms of genetic evolution, physiology, immunology, biochemistry, and pathology, nonhuman primates (NHPs) have outstanding advantages in model construction for disease mechanism study and drug development. In terms of animal model construction, gene editing technology has been widely applied to this area in recent years. This review summarizes the current progress in the establishment of NHPs using gene editing technology, which mainly focuses on rhesus and cynomolgus monkeys. In addition, we discuss the limiting factors in the applications of genetically modified NHP models as well as the possible solutions and improvements. Furthermore, we highlight the prospects and challenges of the gene-edited NHP models.

## Introduction

With the development of human medical research, a broader and deeper understanding of disease pathology and identification of therapeutic strategies has become quite urgent. The establishment of genetically modified animal models is an important aspect of human disease studies. The validity of the animal model is based on its evolutionary similarity to humans. So far, a lot of research has been conducted on the construction of rodent models such as mice models (Gurney, 2000; Wong et al., 2002). Though mice models have played a big role in clinical research, models with higher similarity to humans are needed to study pathogenesis, especially in neurological diseases (Wong et al., 2019). Compared to other animal models, primates are more similar to humans in terms of genetic background and physiological characteristics (Zhao et al., 2019). With up to 93% homologous genome between monkeys and humans, the NHP model has become irreplaceable in studying human diseases (Gibbs et al., 2007; Yan et al., 2011; Higashino et al., 2012; Brasó-Vives et al., 2020).

Though scientists have obtained primate models by screening natural mutations, drug induction, and traditional genetic engineering methods previously (Chan, 2004; Chen et al., 2012; Forny-Germano et al., 2014), it was difficult to obtain by spontaneous mutation. Traditional transgenic methods are not only inefficient but also require many embryonic stem cells, which is time consuming and laborious. In addition, other methods such as retroviral or lentivirus-mediated gene modification methods, RNA interference techniques, and sperm vector-mediated methods are subject to large exogenous random insertion of foreign genes with unstable expression and low operability accompanied by significant chimerism. Until recent years, with the development of gene editing technologies, researchers can use TALEN and CRISPR technology to achieve precise genetic modification on monkeys with the advantage of higher efficiency and accuracy which are unmatched by other methods. Here, we will present recent developments of gene-modified rhesus and cynomolgus monkeys and discuss the factors limiting the application of NHP models and highlight their future applications prospects.

## Traditional transgenic methods to establish NHP models

The classical method of constructing transgenic primates mainly depends on the retrovirus vector-mediated approach, which recombines the target gene into the retroviral vector and integrates the exogenous target gene into the host genome by infecting the host cells. This method has been applied to mice for a long time (Gordon et al., 1980). In 2001, Chan et al. (2001) successfully established a transgenic rhesus monkey termed ANDi by transducing oocytes with high-titer lentivirus followed by sperm injection and embryo transfer, which is the first genetically modified primate in the world. Although the expression of the exogenous gene *GFP* was detected in only one of the three births obtained, this pioneering work marked the birth of transgenic NHP technology. In the same year, another group also constructed the transgenic rhesus monkeys with exogenous *eGFP* integrated into the placental tissue (Wolfgang et al., 2001). Taken together, these two studies provide the basis for the feasibility of exogenous gene integration and lay the foundation for future functional gene studies. Seven years later, scientists successfully obtained the transgenic macaque model expressing the human *HTT* gene to study Huntington’s disease (HD) (Yang et al., 2008). This is the first transgenic primate model with human disease-causing gene integration, which not only helps to study the pathogenic mechanism of HD and explores corresponding treatment options but also makes it possible for studying other genetic diseases such as Parkinson’s disease and Alzheimer’s disease. Subsequently, Sasaki et al. (2009) successfully generated germline-transmissible *GFP* transgenic marmosets through lentiviral vector transfection. For the first time, this study demonstrated germline inheritance of transgenic traits in primates. In addition, people from other countries also established transgenic monkey models (Niu et al., 2010; Liu et al., 2016a).

**FIGURE 1:**
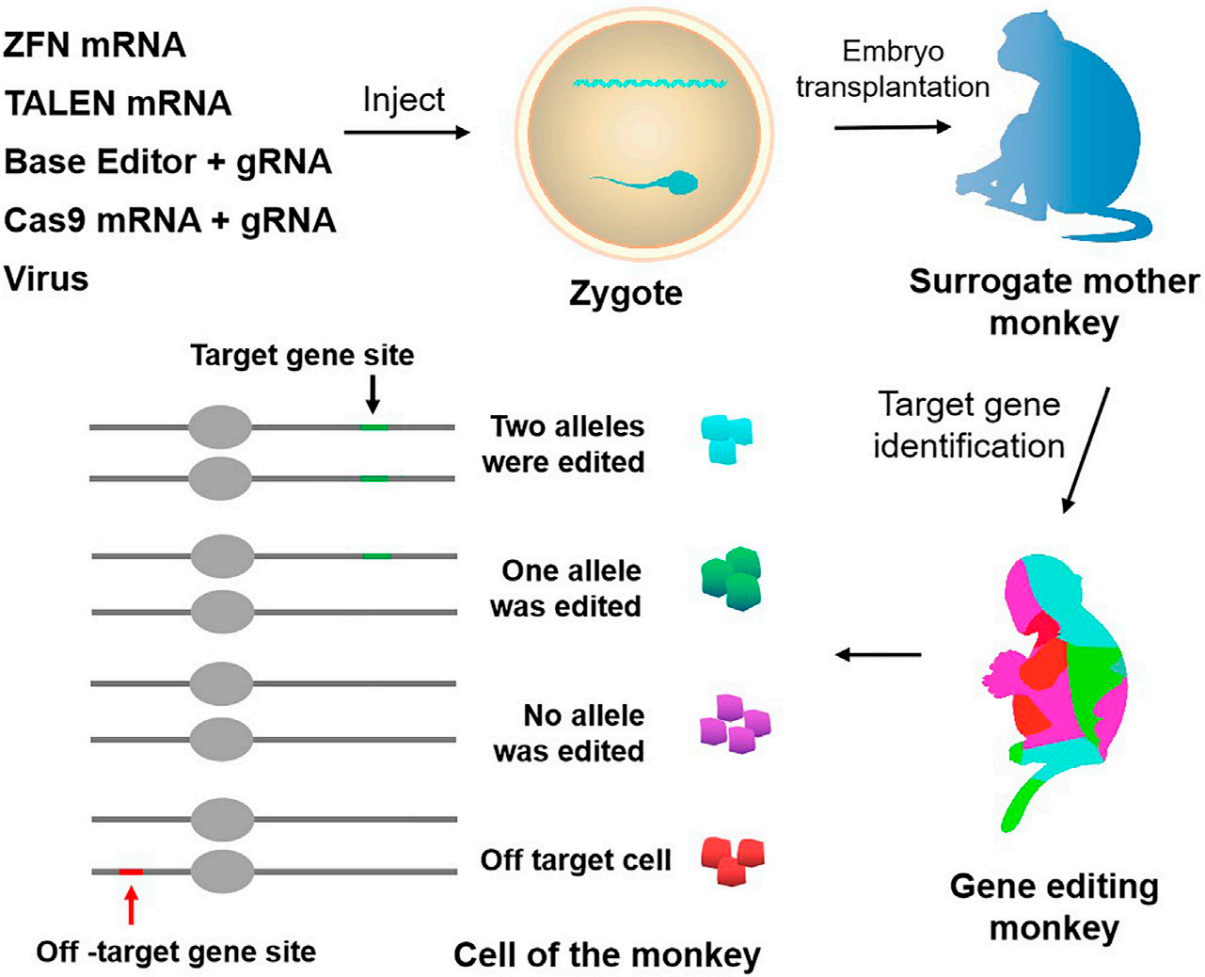
Approaches to genetically modify monkeys. Current techniques to genetically modify monkeys include virus, ZFN, TALEN, CRISPR-Cas9, and base editor. Chimerism exists in gene editing monkeys, and there is the potential for off-targeting.

Although some progress has been made in the construction of NHP models through the retroviral vector methods (Figure 1; Tables 1, 2), these gene modification sites are random and uncertain, and precise genetically modified NHP models cannot be obtained. In addition, the length of the fragment inserted is also limited since lentiviral vectors can only carry fragments no larger than 10 kb, which also brings many uncertainties and limitations to the research. Finally, the low efficiency of transgenic methods also restricts their application. Therefore, more precise and efficient gene editing methods are needed to overcome these limitations.

**TABLE 1:** Rhesus monkey models established by various gene editing technologies.

Modified gene	Method	Success rate	Disease model	Year	References
*GFP*	Retrovirus-mediated gene transfer	20%	None	2001	Chan et al. (2001)
*HTT-84Q, GFP*	Lentivirus-mediated gene transfer	22%	Huntington’s disease	2008	Yang et al. (2008)
*EGFP*	Simian Immunodeficiency Virus (SIV)-based lentivirus-mediated gene transfer	50%	None	2010	Niu et al. (2010)
*MeCP2*	TALEN	67%	Rett syndrome	2014	Liu et al. (2014a)
*Dystrophin*	CRISPR/Cas9	61%	Duchenne muscular dystrophy	2015	Chen et al. (2015b)
*α-Syn*	Lentivirus-mediated gene transfer	85%	Parkinson’s disease	2015	Niu et al. (2015)
*MCPH1*	Lentivirus-mediated gene transfer	100%	Brain development	2019	Shi et al. (2019)
*PINK1*	CRISPR/Cas9	73%	Parkinson’s disease	2019	Yang et al. (2019)
*MeCP2*	CBE	30% (embryo)	Rett syndrome	2020	Qin et al. (2020)

Success rate: number of transgenic monkeys/number of pregnancies (birth).

**TABLE 2:** Cynomolgus monkey models established by various gene editing technologies.

Modified gene	Method	Success rate	Disease model	Year	References
*MeCP2*	TALEN	16.7%	Rett syndrome	2014	Liu et al. (2014a)
*Pparg, Rag1*	CRISPR/Cas9	100%	None	2014	Niu et al. (2014)
*P53*	CRISPR/Cas9	66.7%	*P53* related tumor	2015	Wan et al. (2015)
*Dax1*	CRISPR/Cas9	25%	adrenal hypoplasia congenita, hypogonadotropic hypogonadism	2015	Kang et al. (2015)
*GFP*	Embryo stem cell transplantation	—	None	2015	Chen et al. (2015c)
*MeCP2*	Lentivirus-mediated gene transfer	100%	Rett syndrome	2016	Liu et al. (2016a)
*GFP*	Lentivirus-mediated gene transfer	50%	None	2016	Seita et al. (2016)
*MCPH1*	TALEN	33%	Microcephaly	2016	Ke et al. (2016)
*SHANK3*	CRISPR/Cas9	100%	Autism spectrum disorders	2017	Zhao et al. (2017)
*MeCP2*	TALEN	81%	Rett syndrome	2017	Chen et al. (2017b)
*mCherry*	CRISPR/Cas9	—	None	2017	Yao et al. (2017)
*SIRT6*	CRISPR/Cas9	100%	developmental retardation	2018	Zhang et al. (2018)
*Oct4-GFP*	CRISPR/Cas9	62%	None	2018	Cui et al. (2018)
*SHANK3*	CRISPR/Cas9	55%	Autism spectrum disorders	2019	Zhou et al. (2019)
*BMAL1*	CRISPR/Cas9	62%	Circadian disruption	2019	Qiu et al. (2019)
*PKD1*	CRISPR/Cas9	80%	Autosomal dominant polycystic kidney disease	2019	Tsukiyama et al. (2019)
*HBB*	CRISPR/Cas9	100%	β-Thalassemia	2019	Huang et al. (2019)
*LMNA*	CBE	83%	Hutchinson-Gilford progeria syndrome	2020	Wang et al. (2020)
Multiple targets	CBE, ABE	—	None	2020	Zhang et al. (2020b)
*Pten, p53*	CRISPR/Cas9	87%	Primary and metastatic liver tumors	2021	Zhong et al. (2021)

— means no data for live monkeys.

## ZFN and TALEN gene editing technology to build NHP models

Zinc finger endonucleases (ZFNs) structurally comprise a zinc finger DNA-binding domain and a Fok I DNA-cleavage domain (Carroll, 2011; Wood et al., 2011). Transcription activator-like effector nucleases (TALENs) are gene editing tools made by TAL effector DNA-binding domains and FokI DNA-cleavage domains (Cermak et al., 2011; Wood et al., 2011). After recognizing DNA sequences through DNA-binding domains, the DNA-cleavage domains will target specific DNA sequences to introduce double-strand breaks, which subsequently induce cell repair mechanisms to achieve gene knockout or knock-in by non-homologous end joining (NHEJ) or homologous recombination (HR). The ZFN is time consuming and costly in construction and design, with severe off-target effects. So far, there are no reports on the application of ZFNs on rhesus and cynomolgus monkeys. Compared to ZFNs, TALENs technology is relatively simple to design, less costly, and has high DNA sequence specificity and fewer off-target events, which make it a useful and effective tool to achieve gene modification. Previous studies have witnessed its successful application in many species, including mice, rats, zebrafish, Xenopus, Iberian ribbed newts, zebrafish, and maize (Lei et al., 2012; Qiu et al., 2013; Hayashi et al., 2014; Hisano et al., 2014; Liang et al., 2014; Chen et al., 2017a). Recently, more scientists have started to turn their attention to primates. In 2014, one group successfully generated *MeCP2* gene mutated female cynomolgus monkeys with Rett syndrome (RTT) using TALENs technology (Liu et al., 2014a). RTT cynomolgus monkeys have many similarities with RTT patients in both genotype and phenotype, which is of great significance for human research on the pathogenesis and treatment of RTT. Afterward, other groups also obtained *MeCP2* gene-modified monkeys by using TALENs technology (Liu et al., 2014b; Chen et al., 2017b). In 2016, Ke et al. reported another case that constructed an *MCPH1* gene-modified cynomolgus monkey model mimicking human microcephaly using TALENs-based method (Ke et al., 2016).

In sum, the application of TALENs technology has been widely reported in various studies (Figure 1; Tables 1, 2). However, the gene editing technique requires the design of different recognition proteins for different target sites, which involves huge protein modification work and is time consuming, labor intensive, and costly. These limitations seriously restrict the wide application of TALENs.

## Application of CRISPR/Cas9 gene editing technology in NHPs

The CRISPR/Cas system, which is short for clustered regularly interspaced short palindromic repeats and CRISPR-associated protein, was originally characterized as a defense mechanism that bacteria use against viruses (Horvath and Barrangou, 2010). It was subsequently engineered to cleave DNA in eukaryotes (Cong et al., 2013; Mali et al., 2013). Due to its simplicity, efficiency, and technical flexibility, CRISPR/Cas technologies became strong tools in biological and biomedical studies of various cell types and living organisms.

As for primates, there are also gratifying results. In 2014, for the first time, the researchers obtained twin cynomolgus monkeys carrying targeted mutation genes and achieved simultaneous knockout of two genes *PPARγ* and *RAG1* by using CRISPR/Cas9 technology (Niu et al., 2014), and it’s important for studying immune system related diseases. This was the first application of CRISPR gene editing technology in primates. More importantly, this study achieved the simultaneous knockout of two target genes in one step. In addition, the DNA samples extracted from the umbilical cord and placenta of newborn monkeys were detected and analyzed, and no off-target phenomenon was found. Finally, they also found the mutation appeared in germ cells, which demonstrated that genetic mutations mediated by CRISPR technology can enable germline transmission (Chen et al., 2015a). The success of this study makes it possible to establish animal models of some complex diseases controlled by multiple genes. Duchenne muscular dystrophy (DMD) is a genetic disorder characterized by muscle degeneration due to the mutant muscle protein dystrophin. In order to study this disease, Chen et al. generated mutant rhesus monkeys by using CRISPR/Cas9 technology to target the dystrophin gene (Chen et al., 2015b). They analyzed the muscle tissue of monkeys who died of dystocia, finding that the expression of dystrophin protein was significantly decreased, which was similar to that of DMD patients. And the degeneration of muscle cells at an early stage was observed in both monkey models and DMD. In the same year, one group obtained a *P53* gene (tumor suppressor gene) biallelic mutant cynomolgus monkey model without germline mating by optimizing the CRISPR/Cas9 technology (Wan et al., 2015). Meanwhile, another group generated a cynomolgus monkey model with *DAX1* gene deletion via CRISPR/Cas9 technology (Kang et al., 2015). This *DAX1* mutant monkey exhibited adrenal developmental defects and aberrant testicular architecture which were very similar to the clinical features of AHC-HH patients. Another study reported a rhesus monkey model with hemoglobin beta gene modified by CRISPR/Cas9 technology, and they found a way to improve the gene editing efficiency and reduced unfavorable outcomes such as off-target effects by optimization of sgRNAs concentrations (Midic et al., 2017). In 2019, scientists utilized CRISPR/Cas9 to establish *SHANK3* gene mutated cynomolgus macaques and their F1 offspring (Zhou et al., 2019), which could lead to sleep disorders, movement disorders, increased repetitive behaviors, and social and learning disabilities similar to what happened in autism spectrum disorders. Another example is the *SIRT6* gene, the scientists successfully generated *SIRT6* gene-modified cynomolgus monkeys, which showed a deficiency in *SIRT6* function (Zhang et al., 2018). SIRT6 has been identified as a longevity protein in mice (Mostoslavsky et al., 2006). As expected, the model monkey died shortly after birth and displayed severe developmental retardation. A recent study reported a new method to generate gene-modified monkeys by *in situ* CRISPR-mediated technique (Zhong et al., 2021). This study utilized CRISPR/Cas9 to knock down *Pten* and *p53* genes in adult cynomolgus, thus modeling primary and metastatic liver tumors rapidly, which was effective *in situ* gene editing approach. Other groups also generated gene-edited monkeys using this method (Figure 1; Tables 1, 2).

## Limitation factors affecting the application of gene-edited NHP models

Whether it is transgenic monkeys obtained by lentiviral vector transduction or gene-edited monkeys generated by targeting nucleases, most of the founder monkeys exist in the form of chimeras. For surviving chimeric founder monkeys, it is difficult to precisely map the transgene or target gene mutation to the underlying phenotype. When using lentiviral vectors to construct transgenic monkeys, various numbers of lentiviral vector sequences can be integrated into the monkey genome at different time points in early embryos, accompanied by the randomness of integration numbers and time. This leads to the possibility that the number of transgene copies and the transgene integration sites of individuals obtained from different embryos injected with the same batch of viral vectors may vary greatly, and the number of transgenes and integration sites contained in different cells of the same transgenic individual may also be different (Liu et al., 2016a). Then the same batch of transgenic founder monkeys obtained by the same lentivirus may have phenotypic differences. To overcome this problem, a non-integrating lentiviral vector (NILV) has been developed by integrase mutation to reduce the risks of random insertion (Wanisch and Yáñez-Muñoz, 2009). Currently, NILVs have shown efficacy in different preclinical mice models, such as Parkinson’s disease and Hemophilia B, with relatively lower expression levels than integrating lentiviral vectors though (Lu-Nguyen et al., 2014; Suwanmanee et al., 2014). So, whether NILV can construct transgenic NHP models with fewer random insertion risks still remains to be seen.

Similarly, when using targeted nucleases such as ZFN, TALENs, and CRISPR/Cas9 to construct gene editing monkeys, the founder monkeys obtained always exist in the form of chimeras (Niu et al., 2014; Chen et al., 2015b; Guo and Li, 2015) (Figure 1). Due to the long growth cycle of NHPs, it takes a lot of time and money to screen animal models with purely targeted gene modification through multi-generational mating. Chimeric mutations can also seriously affect the functional studies of target genes and the pathological analysis of related diseases, so it is particularly necessary to reduce the generation of chimeras. Two main reasons account for chimeric mutation. First, after Cas9 modifies the target site, the DNA repair activity between dividing cells may differ, resulting in different degrees of mutation of the target gene between different cells and tissues. Second, random insertion and deletion (Insertion/deletion, indel) will occur in the process of the NHEJ-mediated DNA repairing, resulting in various indels at the target site, leading to the formation of multi-genotype chimeric mutants.

Another problem in constructing gene editing monkeys using targeted nucleases such as ZFNs, TALENs, and CRISPR/Cas9 is off-targeting (Figure 1; Tables 3, 4). Previous studies did by multiple groups showed significant off-target activity in the CRISPR/Cas9 system (Fu et al., 2013; Pattanayak et al., 2013; Cho et al., 2014). Although no off-target effects have been found in NHPs models built by the CRISPR/Cas9 system which may be due to the limitation of the number of detection sites, it is difficult to conclude that these founder monkeys did not have off-target mutations. Therefore, the existence of the off-target phenomenon is also one of the unavoidable factors that potentially limit the in-depth study of gene editing monkeys as animal models.

**TABLE 3:** Comparison of the three main used genome editing technologies in monkeys: ZFN, TALEN, and CRISPR.

	ZFN	TALEN	CRISPR
Target recognition	Protein-DNA	Protein-DNA	RNA-DNA
Number of target sequence (bp)	18–36 bp	24–40 bp	∼23 bp
Sequence recognition	3 bp as a unit	Requires a T at 5′-end of the target sequence	Requires NGG sequence at 3′-end
Build difficulty	Difficult	Easy	Very easy
Editing RNA	No	No	Yes
Off-target	High	High	Low
Cytotoxicity	High	Low	Low

**TABLE 4:** The advantage and disadvantages of the three main used gene editing technologies.

	ZFN	TALEN	CRISPR
Advantage	Mature technology	High specificity, simpler design than ZFN, high success rate	Low off-target effects, low cytotoxicity, cheap
Disadvantage	Low success rate, high off-target effects, high cytotoxicity	Cumbersome process, heavy workload, high cost	The possibility of off-target

The third problem is the low efficiency of gene knock-in. The incidence of HR repair for gene editing is very low in the current use of the CRISPR/Cas9 system for NHPs. The only successful results are the knock-in of small DNA sequences (Wan et al., 2015). However, no successful knock-in of large segments of gene sequences has been reported in NHPs.

## Refinement of strategies to overcome these deficiencies

All the factors mentioned above strictly limited the broad application of NHPs as animal models for human diseases. So, there is an urgent need for some improvements to overcome these deficiencies. With the development of technology, some effective methods start to appear. As for chimerism, current researches show that the continuous expression of Cas9 protein may increase chimeric mutation. So, it is easy to imagine that restricting Cas9 expression only at the one-cell stage may be an effective method to reduce chimeric mutation. One group established a new method in which they linked ubiquitin-proteasome to the N-terminus of Cas9 protein, which can facilitate the degradation of Cas9 protein in cynomolgus monkey embryos whereby reducing the mosaic mutations (Tu et al., 2017). Similarly, other ways of controlling the Cas9 activity can also be applied in the future establishment of gene-edited NHPs models such as small molecules, light, inhibitors, and degraders (González et al., 2014; Nihongaki et al., 2015a; Nihongaki et al., 2015b; Zhou et al., 2018; Gangopadhyay et al., 2019; Maji et al., 2019). Another alternative approach to avoid mosaic mutations is the F1 offspring. As is well known, no matter what form of chimerism it is in the founder monkey, the F1 offspring can only get a specific edited genotype. Since the sexual maturation cycle of NHPs is very long, which takes about 4–5 years to reach sexual maturity for commonly used rhesus and cynomolgus monkeys, the development of methods to shorten the sexual maturation cycle of NHPs to achieve accelerated reproduction will promote the application of NHPs research. For this purpose, one group has developed testis xenotransplantation to accelerate spermatogenesis. By transplanting juvenile cynomolgus monkey testis tissue blocks to the back of adult male nude mice, the spermatogenesis time of cynomolgus monkeys was successfully shortened to 24 months, and the obtained sperm was used for embryo construction and transplantation to obtain healthy offspring of cynomolgus monkeys (Liu et al., 2016b). Therefore, the development of other methods to accelerate the reproduction cycle of NHPs will also be an important research direction for future NHP genetic modification models. Finally, one group generated complete gene knockout monkeys in one step by multiple sgRNAs, which provides another method to reduce the chimerism rate (Zuo et al., 2017).

In addition, for the off-target phenomenon, some improvements have been made to avoid this. One method is to screen and predict potential off-target sites in silico and optimize the gRNA design accordingly to minimize the off-target effects. Sangsu et al. developed a tool termed Cas-OFFinder, which can search for potential off-target sites in a given genome or user-defined sequences (Bae et al., 2014). Another method is Cas9 protein optimization. People from various groups have utilized different ways to optimize the Cas9 protein including fusion protein and mutated protein to achieve lower or no off-target efficiency (Koo et al., 2015; Anders et al., 2016; Kleinstiver et al., 2016; Slaymaker et al., 2016). In addition, controlling Cas9 protein expression can be used as another strategy to circumvent the off-target. Some groups have used inducible Cas9 protein and Cas9 inhibitory protein to achieve this goal (Nihongaki et al., 2015a; Rauch et al., 2017). All these methods are worth trying on monkeys in the future.

As for low knock-in efficiency, there are also some promising results. In principle, two ways can be used to improve the knock-in efficiency, one is to block non-homologous end joining, and the other is to increase homologous repair. Previous studies have shown that Scr7 which can bind to Ligase IV, a key component in the NHEJ process, can be used to block the NHEJ pathway whereby improving the precise repair (Chu et al., 2015; Vartak and Raghavan, 2015; Maruyama et al., 2016). In addition, another group enhanced the HR efficiency by optimizing donor DNA (Richardson et al., 2016).

Lastly, base editor systems can also be used to achieve precise gene editing, by which one base can be converted to another base precisely without requiring DNA cleavage, thus decreasing insertion or deletion events that happened randomly at the specific sites caused by DSB.

Single-base editing technology mainly realizes gene editing through the complex formed by deaminase, Cas9 variant, and sgRNA, in which sgRNA is responsible for guiding the complex to target the target sequence, and deaminase is responsible for deamination to achieve single-base editing (Komor et al., 2016; Gaudelli et al., 2017). According to different editing sequences and editors, the developed single-base editing technologies can be divided into two categories, namely cytosine base editing (CBE) technology and purine base editing (ABE) technology. CBE technology mainly realizes C→T or G→A conversion through sgRNA, inactive Cas9 protein (dCas9), and cytosine deaminase (Komor et al., 2016), there are various types of CBEs such as CBE1, CBE2, CBE3, CBE4, HF-CBE, SaBE4, CBE4-Gam, eCBE et al. ABE technology mainly realizes A→G or T→C base editing through sgRNA, adenosine deaminase and Cas9n, in which sgRNA is responsible for guiding the complex to target the target sequence, and adenosine deaminase is responsible for catalyzing the deamination of adenine in the target sequence (Gaudelli et al., 2017). Various types of ABEs have been developed such as xABE, SaABE, VRER-ABE, ScCas9-ABE, et al. Until now, there are different types of base editor systems developed and applied (Komor et al., 2016; Gaudelli et al., 2017; Komor et al., 2017; Koblan et al., 2018; Molla and Yang, 2019; Zhang et al., 2020a). In addition, point mutation is a key cause of genetic diseases. Therefore, the base editor systems can be used in constructing gene-edited monkeys mimicking the diseases caused by point mutations. One group successfully generated base-edited cynomolgus monkeys with multiple target sites simultaneously modified using cytidine- and adenine-base editors (Zhang et al., 2020b).

## Conclusion and perspectives

**FIGURE 2:**
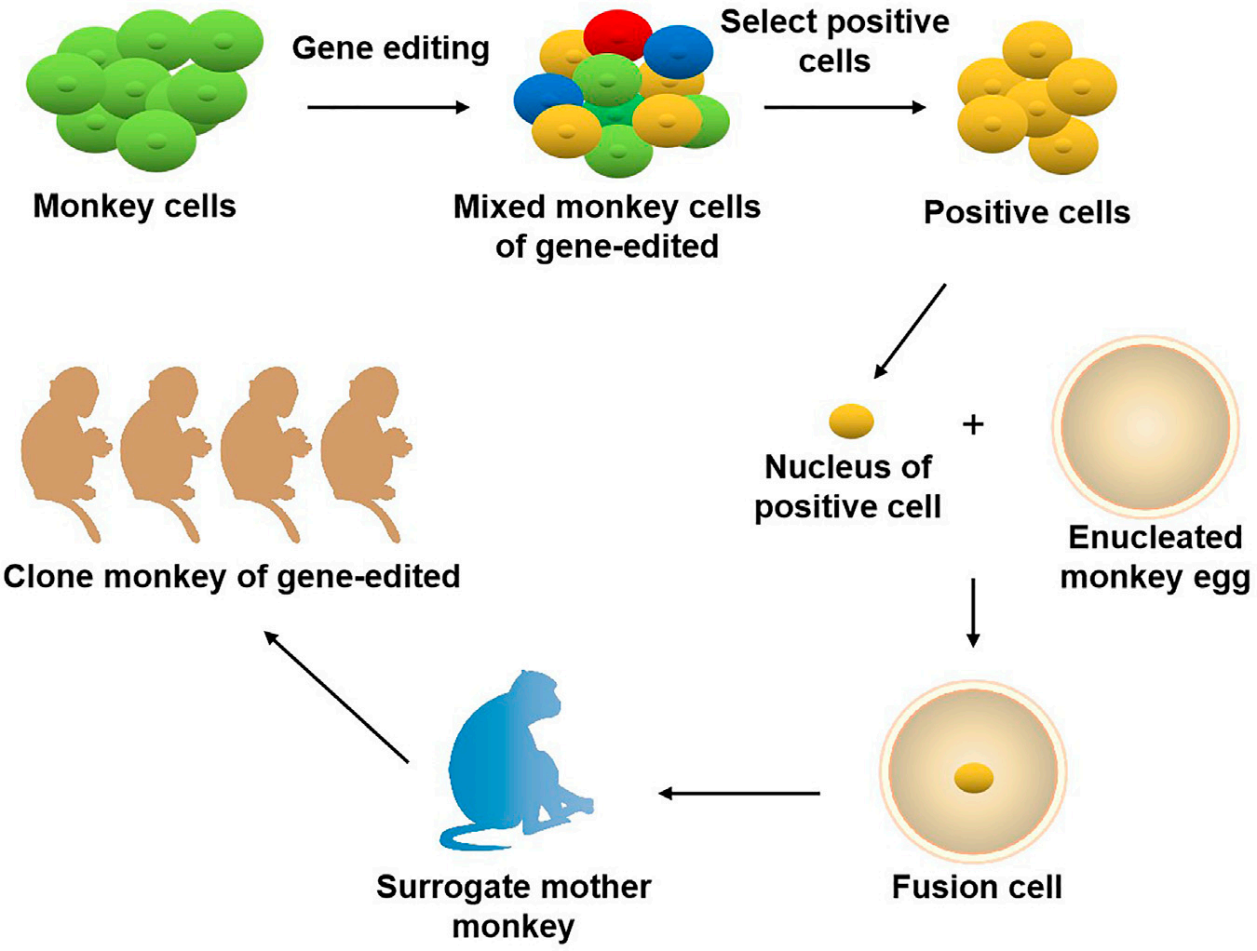
Joint application of gene editing and nuclear transfer technique in producing gene-modified monkeys. Using gene editing technology to modify the genome of monkey cells, and screen out specific types of positive cells. The nuclei of the positive cells are transferred to the enucleated monkey eggs, and the fusion cells are transferred to the surrogate mother monkey to obtain the gene-edited cloned monkeys.

**FIGURE 3:**
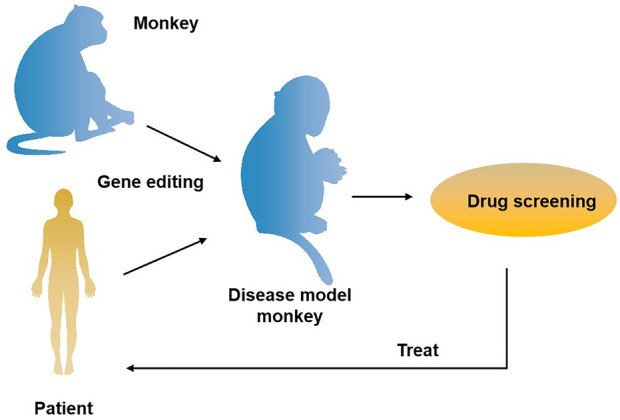
The application of gene-edited monkey models in drug development and treatment for the disease. A transgenic monkey model constructed by gene editing can be applied to drug screening for human diseases such as neurodegenerative diseases and cancer, thus providing an opportunity for disease treatment.

The role primates play in the field of biomedical research is unmatched by other species. It is well known that the odds for a newly discovered drug to come to the market are lower than 10%. The primary cause for this situation is that there are no ideal animal models available that mimic human diseases, such as cancer. Currently, two animal models are commonly used clinically for drug and vaccinee valuation in the antitumor market, which in rodents and NHPs. The former is preferred by many scientists due to its short reproduction cycle, cheap, small, clean genetic background, and genetic operability. However, the large species divergences between humans and rodents render many failures in clinical trials though the drug has shown satisfying safety and efficacy in preclinical trials. NHPs, however, such as cynomolgus monkeys and rhesus monkeys, display considerable similarities to humans in terms of genetic background, physiological composition, and immunological nature. As such, NHP has an advantage over other animal models to be applied in drug research and development and preclinical animal trials for safety and efficacy evaluation. Monkeys and chimpanzees share many cancer genes with humans. However, they are seldomly used in cancer research and drug development because of high costs and ethical issues. Furthermore, generating loss-of-function or gain-of-function mutations in NHPs by breeding remains cumbersome and challenging compared to rodents due to their longer sexual maturity cycle. On the other hand, using nuclear transfer (NT) technology to obtain cloned transgenic animals is the most direct and reliable method, which is a very mature technology in some species (Zhou et al., 2015). One group successfully obtained healthy cloned monkeys using somatic cell nuclear transfer technology, which provides important technical support for gene editing and model construction of NHPs (Liu et al., 2018). Therefore, combining gene editing technology with nuclear transfer and other technologies in the future is expected to contribute to constructing primate disease models more efficiently (Figure 2). Meanwhile, with the continuous updating and improvement of technology, the establishment of primate models of human diseases will eventually provide more possibilities for scientists to deeply study disease mechanisms and explore new disease treatments, which will eventually bring a boon to human health (Figure 3).
